# Effects of a Single Sub-Anesthetic Dose of Ketamine on Postoperative Emotional Responses and Inflammatory Factors in Colorectal Cancer Patients

**DOI:** 10.3389/fphar.2022.818822

**Published:** 2022-04-05

**Authors:** Qin Ren, Ling Hua, Xiaofang Zhou, Yong Cheng, Mingjun Lu, Chuanqing Zhang, Jianrong Guo, Hua Xu

**Affiliations:** ^1^ Department of Anesthesiology, Shanghai Pudong New Area Gongli Hospital, Navy Military Medical University, Shanghai, China; ^2^ Department of Laboratory Medicine, Shanghai Pudong New Area Gongli Hospital, Navy Military Medical University, Shanghai, China; ^3^ Department of Anesthesiology, Yueyang Hospital of Integrated Traditional Chinese and Western Medicine, Shanghai University of Traditional Chinese Medicine, Shanghai, China

**Keywords:** ketamine, sub-anesthetic dose, depression, anxiety, inflammatory factors

## Abstract

**Objective:** To investigate the effect of a single sub-anesthetic dose of ketamine on postoperative anxiety, depression, and inflammatory factors in patients with colorectal cancer.

**Methods:** A total of 104 patients undergoing selective colorectal surgery in our hospital from Jan 2015 to Oct 2017 were included and randomly assigned (1:1:1:1) into a 0.1 mg kg^−1^ ketamine group (K1 group), 0.2 mg kg^−1^ ketamine group (K2 group), 0.3 mg kg^−1^ ketamine group (K3 group), or control group (C group). Corresponding doses of ketamine were given intravenously in the K groups (K1, K2, and K3 groups) 5 min before operation, and the same amount of normal saline was given in the C group. The intravenous analgesia program was identical in the four groups. The patients’ emotional reactions (anxiety and depression) were assessed by the Hospital Anxiety and Depression Scale (HAD), the quality of postoperative recovery was evaluated by the Quality of Recovery-40 (QoR-40) questionnaire, and the levels of IL-6, IL-8, and TNF-α in peripheral blood were detected by enzyme-linked immunosorbent assay (ELISA) on the day before operation and within 24, 48, and 72 h post-operation respectively. Pain was estimated by the visual analog scale (VAS), and sedation was assessed with Ramsay score 30 min after extubation. The time points of anesthetic end and extubation were recorded. The complications during anesthesia and recovery such as cough and agitation 30 min after extubation were recorded.

**Results:** The anxiety score (HAD-A) and depression score (HAD-D) of the K3 group were significantly lower than those of the C group post-operation (*p* < 0.05). The QoR-40 score of the K3 group was significantly higher than that of the C group (*p* < 0.05). The serum levels of IL-6, IL-8, and TNF-α in the K3 group were significantly lower than those in the C group (*p* < 0.05 and *p* < 0.01). There were no significant differences in HAD-A, HAD-D, and QoR-40 scores or serum levels of IL-6, IL-8, and TNF-α between the K1 and K2 groups and the C group. There were no significant differences in VAS pain score or Ramsay sedation score among the four groups 30 min after extubation. There were no significant differences in extubation time, postoperative cough, emergence agitation, or delirium among the four groups. Dizziness, nausea, vomiting, diplopia, or other adverse reactions were not found 30 min after extubation.

**Conclusion:** A single sub-anesthetic dose (0.3 mg kg^−1^) of ketamine can significantly improve the postoperative anxiety and depression of colorectal cancer patients and reduce the levels of IL-6, IL-8, and TNF-α.

## Introduction

Postoperative depression is a kind of mental disorder caused by various factors during perioperative period, and it is characterized by significant and lasting depression (Ghoneim and O’Hara, 2016). It was reported that the incidence of depression/anxiety in colorectal cancer patients after operation was as high as 66.7% (Medeiros et al., 2010; Cardoso et al., 2016), which seriously affected the perioperative treatment, the quality of recovery, and even the long-term outcome (Medeiros et al., 2010; Miniotti et al., 2019). There was increasing evidence that depression was closely related to immune inflammatory factors (Haapakoski et al., 2016; Aminisani et al., 2017; Coskun et al., 2017). Surgery and stress can activate the peripheral immune system, leading to the release of inflammatory mediators and cytokines, which may induce postoperative depression (Andersson, 2013). Ketamine, an N-methyl-D-aspartate (NMDA) receptor antagonist, can inhibit the medial thalamic nucleus, block the ascending conduction of reticular formation of the spinal cord tract, and excite the limbic system (Liebe and Meng, 2018). With its strong analgesic effect, it is a kind of non-barbital intravenous general anesthetic often used in clinics. Recent clinical studies found that ketamine, with a rapid onset of antidepressant effect, was the only drug that induces significant antidepressant response in patients immediately after a single administration (Ionescu et al., 2015).

Compared with traditional antidepressant drugs, the most obvious advantage of ketamine was the usually rapid onset of action. Two hours after the intravenous injection of ketamine, the Hamilton depression score (HAMD) of patients with severe depression began to decline. Within 24 h after treatment, more than 70% of the patients reached the standard response (with 50% improvement), and 35% remained effective after 1 week (Ibrahim et al., 2010). The duration of the antidepressant effect of ketamine was much longer than its half-life. No other single drug has ever had such a long-lasting effect. At the same time, the results of a large number of animal experiments have also shown that ketamine had rapid and effective antidepressant effect in a variety of depression models (Yan et al., 2010; Zhou et al., 2015). However, it is not well known whether ketamine has therapeutic effect on depression/anxiety after surgery or whether it can affect postoperative inflammatory factors in clinic. The purpose of this study is to explore the therapeutic effect of sub-anesthetic dose of ketamine on postoperative emotional responses and the influence on the levels of inflammatory factors.

## Materials and Methods

### General Information

A randomized, double-blind, placebo-controlled trial was designed, and the study protocol was approved by the medical ethics committee of Gongli hospital, Navy Medical University (Second Military Medical University), Shanghai (No. 2014-15). Patients undergoing selective colorectal surgery in Gongli hospital from Jan 2015 to Oct 2017 were included. All of them gave their signed informed consent before inclusion. Inclusion criteria were as follows: 1) patients with American Society of Anesthesiologists (ASA) class I–II identification undergoing elective colorectal cancer surgery under general anesthesia for less than 4 h; 2) the incision was expected to be more than 10 cm; and 3) patients aged from 40 to 70 years with the body mass index (BMI) ranged from 18 to 24 kg m^−2^. Exclusion criteria were as follows: 1) patients with poor understanding and mental or central nervous system disorders before operation; 2) patients with diabetes and heart disease; 3) patients with hormone therapy during operation; 4) patients with ketamine or opioid allergy; 5) patients with severe liver and kidney dysfunction; and 6) patients with alcohol addiction or frequent use of sedative and analgesic drugs.

Power Analysis and Sample Size (PASS) 15.0 was applied to calculate the sample size. In this study, we take Hospital Anxiety and Depression Scale (HAD) depression score (HAD-D) differences between pre-operation and 48 h post-operation as the primary treatment effect index, and we set the inspection level (α) as 0.05 and the test power as 0.8; then, *β* = 1–0.8 = 0.2, and the mean value of HAD-D scores difference was 4.5 with standard deviation 5; for two-sided tests, at least, 23 patients would be required for each group, and considering the rate of loss during follow-up was 10%, 26 patients were observed in each group, which combined up to 104 patients in four groups as total. Then, a computer-generated 1:1:1:1 block randomization scheme in SAS 9.0 software (SAS Institute, Cary, North Carolina) was used to assign patients to the 0.1 mg kg^−1^ ketamine group (K1 group), 0.2 mg kg^−1^ ketamine group (K2 group), 0.3 mg kg^−1^ ketamine group (K3 group), or control group (C group) by a biostatistician who was masked to the data management and statistical analysis of this study. The drug numbers were generated by the block randomization method, and the selected block length and random parameters were stored together as confidential data.

### Anesthesia Methods

Corresponding doses (0.1, 0.2, 0.3 mg kg^−1^) of ketamine (Fujian Gutian Pharmaceutical Co., Ltd., No. 111207) were given intravenously in the K groups (including K1, K2, and K3 groups) 5 min before operation, respectively, while the same amount of normal saline was given in the C group. There were no significant differences in gender ratio, height, weight, or age among the four groups.

All patients were fasting for more than 8 h, with water deprivation for more than 6 h, without any preoperative medication before operation. Informed consent was signed 1 day before the operation and the study was carried out in accordance with the relevant provisions of the hospital ethics committee to ensure the rights and privacy of patients. Meanwhile, the researchers explained the meaning and methods of the HAD, the Quality of Recovery-40 (QoR-40) questionnaire, and the visual analog scale (VAS) to patients. The multi-function monitor was used to monitor the non-invasive blood pressure (NIBP), pulse oxygen saturation (SpO_2_), electrocardiogram (ECG), and end-expiratory carbon dioxide partial pressure (PetCO_2_) in the operation room. Ketamine and normal saline were put into syringes with the same appearance by researchers, and all of them were diluted to 5 ml with normal saline, which was masked to the doctors and patients.

General anesthesia was induced sequentially by intravenous administration of midazolam 0.05 mg kg^−1^, propofol 2 mg kg^−1^, sufentanil 0.5 μg kg^−1^, muscle relaxant cisatracurium 0.2 mg kg^−1^. Endotracheal intubation was completed 3 min after administration, breathing was controlled by ventilator (RR = 12 beats/min, VT = 8 ml kg^−1^), and end tidal carbon dioxide (PetCO_2_) was maintained at 30–35 mmHg. Anesthesia was maintained with 1%–3% sevoflurane (1–2 MAC values, up to 2.5 MAC values), continuous micro-pump infusion of propofol 6–8 mg kg^−1^ h^−1^, remifentanil 0.1–0.2 μg kg^−1^ min^−1^, cisatracurium 5 mg h^−1^, and intermittent addition of sufentanil 10–20 µg. The dosage of anesthetics was adjusted according to the patient’s blood pressure, heart rate, and bispectral index (BIS). Intraoperative blood pressure was maintained at ± 20% of baseline. In case of hypertension (mean arterial pressure > 20% of the basic value), appropriate amount of Urapidil hydrochloride should be applied. In case of hypotension, intravenous infusion should be accelerated or appropriate amount of ephedrine should be applied. Appropriate esmolol should be used when the heart rate >110 bpm. K groups were injected intravenously 5 min before skin incision, while the C group was given the same volume of normal saline. Sufentanil 0.1 μg kg^−1^ was given at the time of suturing skin, and then the patient-controlled intravenous analgesia pump was connected. Extubation was performed when extubation indications were available.

All the patients in the four groups were given patient-controlled intravenous analgesia (PCIA), and the formula was: sufentanil 100 µg + flurbiprofen axetil 200 mg + normal saline diluted to 120 ml, background dose was 2 ml/h, PCA dose was 2 ml, locking time was 20 min, and analgesia time was from the end of operation to 48 h after operation.

Extubation indications: spontaneous breathing recovery, stable circulation, normal tidal volume and minute ventilation volume, oxygen saturation of inspiratory air pulse >95% and lasting for more than 5 min, normal swallowing reflex or cough reflex, call for response, can open eyes or complete mandatory action.

### Clinical Observation Indexes

All the data were collected by medical staff who were masked to the group allocation and the anesthesia process. The meaning and method of HAD, the QoR-40 questionnaire and the VAS were explained to the patients 1 day before the operation. All patients were scored with HAD scale and QoR-40 questionnaire, the HAD anxiety score (HAD-A) and HAD-D were recorded, and the levels of IL-6, IL-8, and TNF-α were detected by enzyme-linked immunosorbent assay (ELISA) pre-operation and 24, 48, and 72 h post-operation. VAS pain score and Ramsay Sedation score were recorded 30 min after extubation. Sufentanil (3–5 µg) can be given to patients with moderate-to-severe pain (VAS pain score >4) or who want analgesia subjectively until they no longer need analgesic drugs. The time from the end of anesthesia to extubation, cough during extubation, delirium during recovery, sedation within 30 min after extubation, dizziness, nausea, vomiting, diplopia, hallucination and other adverse reactions were recorded.

The primary efficacy indexes were the changes of HAD-D 24, 48, and 72 h post-operation compared with the baseline. The secondary efficacy indexes included the changes of HAD-A, QoR-40 and the serum levels of IL-6, IL-8, and TNF-α 24, 48, and 72 h post-operation compared with the baseline, and VAS pain score and Ramsay Sedation score 30 min after extubation.

### Statistical Analysis

SAS 9.3 (SAS Institute, Cary, North Carolina) was used to analyze the data. The analysis included the actual number of subjects enrolled in the four groups, lost and excluded subjects, demographics and other baseline characteristics, compliance, efficacy, and safety. Qualitative data was expressed as numbers and percentages or composition ratios, and analyzed using the chi-square tests, Fisher’s exact tests, and Wilcoxon rank sum tests. Quantitative data was expressed as mean and standard deviation values or as median values with IQR (interquartile range). ANOVA (analysis of variance) was used for quantitative variables that conform to normal distribution, and non-parametric test was used for quantitative variables conforming to skewed distribution, repeated measure ANOVA was used for data with over 2 times of repeated measures. In this study, a two-sided *p*-value of less than 0.05 was considered as statistically significant.

## Results

### Comparison of the General Characteristics

**FIGURE 1:**
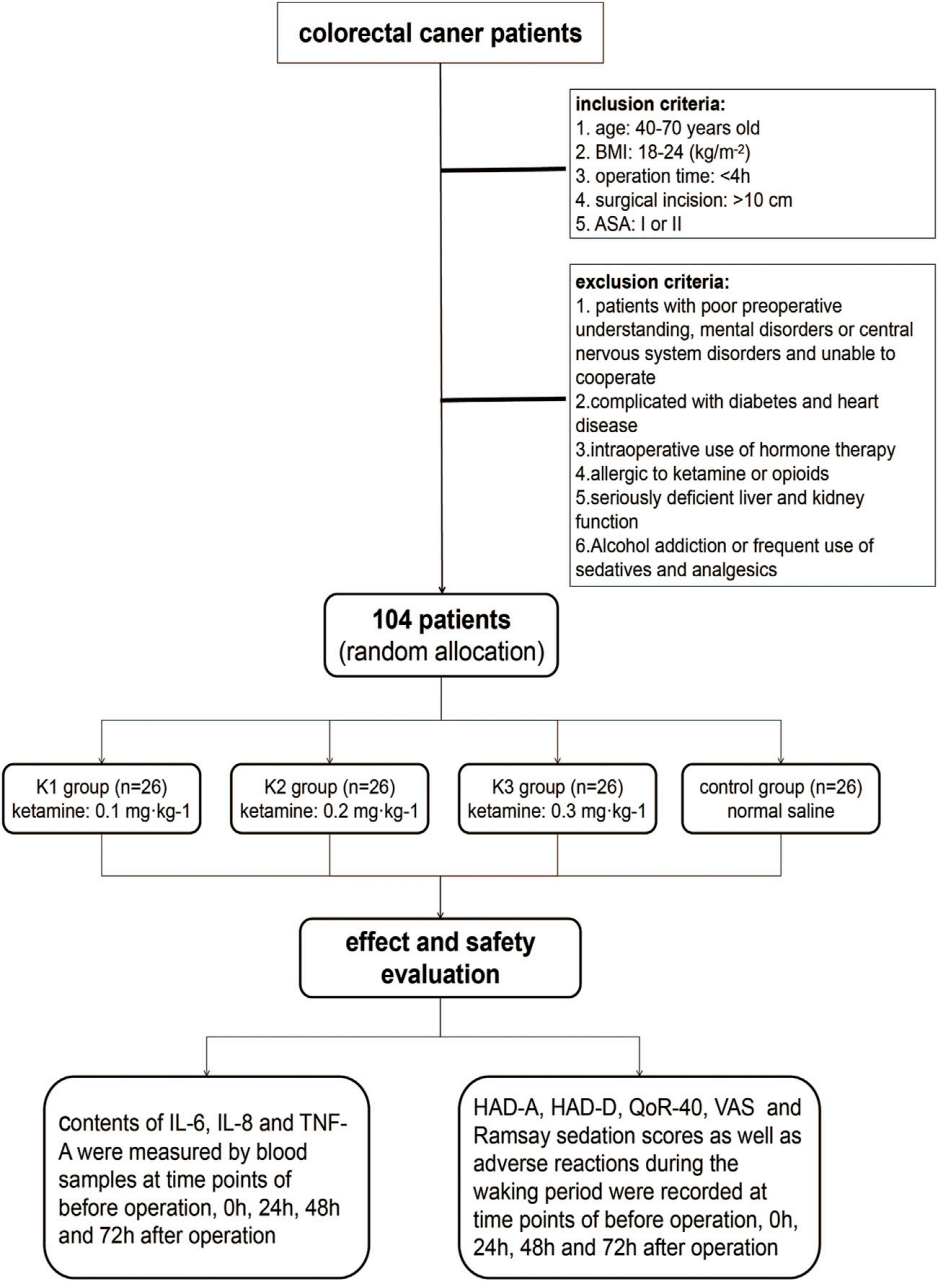
Patient enrollment flowchart. BMI, body mass index; ASA, American Society of Anesthesiologists; HAD, Hospital Anxiety and Depression Scale; HAD-A, anxiety score of HAD; HAD-D, depression score of HAD; QoR-40, Quality of Recovery-40; VAS, visual analog scale.

A total of 104 patients were included in the study, including 3 K groups (K1, K2, and K3 groups) and a control group (C group), with 26 patients in each group. The flow chart of the study is shown in Figure 1. The age of the four groups was 60.8 ± 5.8, 61.3 ± 8.7, 60.4 ± 7.8, and 62.9 ± 6.0, respectively. The body weight of the four groups was 65.1 ± 11.3, 64.2 ± 9.2, 63.7 ± 12.5, and 65.3 ± 10.4, respectively. The male ratio was 46.2%, 53.8%, 50%, and 53.8%, respectively. There were no significant differences in the baseline characteristics among the four groups (all *p* > 0.05) (Table 1).

**TABLE 1:** Demographic character of colorectal cancer patients.

Variables	K1 group (*n* = 26)	K2 group (*n* = 26)	K3 group (*n* = 26)	Control group (*n* = 26)	Chi-square	*p* value
Age (years), median (IQR)	62.0 (58.5–64.3)	63.5 (58.0–67.3)	62.5 (52.0–67.3)	63.5 (61.0–67.0)	2.488	0.478
Gender, n (%)					0.423	0.935
Male	12 (46.2)	14 (53.8)	13 (50.0)	14 (53.8)		
Female	14 (53.8)	12 (46.2)	13 (50.0)	12 (46.2)		
Weight (kg), median (IQR)	62.5 (59.5–70.0)	65.0 (60.0–70.0)	61.5 (55.0–73.0)	67.5 (57.3–73.5)	0.507	0.917
BMI, median (IQR)	23.4 (21.4–23.9)	23.3 (20.9–23.7)	22.8 (20.3–23.8)	23.2 (21.5–23.7)	1.908	0.592
Accompanied diseases, n (%)					0.975	0.807
Yes	17 (65.4)	16 (61.5)	14 (53.8)	17 (65.4)		
No	9 (34.6)	10 (38.5)	12 (46.2)	9 (34.6)		
ASA level, n (%)					1.611	0.657
I	4 (15.4)	4 (15.4)	7 (26.9)	6 (23.1)		
II	22 (84.6)	22 (84.6)	19 (73.1)	20 (76.9)		
Stage of cancer, n (%)					6.449	0.092*
T1	4 (15.4)	4 (15.4)	5 (19.2)	2 (7.7)		
T2	9 (34.6)	9 (34.6)	6 (23.1)	3 (11.5)		
T3	3 (11.5)	3 (11.5)	2 (7.7)	3 (11.5)		
T4	10 (38.5)	10 (38.5)	13 (50.0)	18 (69.2)		

### Comparison of Clinical Indexes

#### Changes of HAD-A in the Four Groups

**FIGURE 2:**
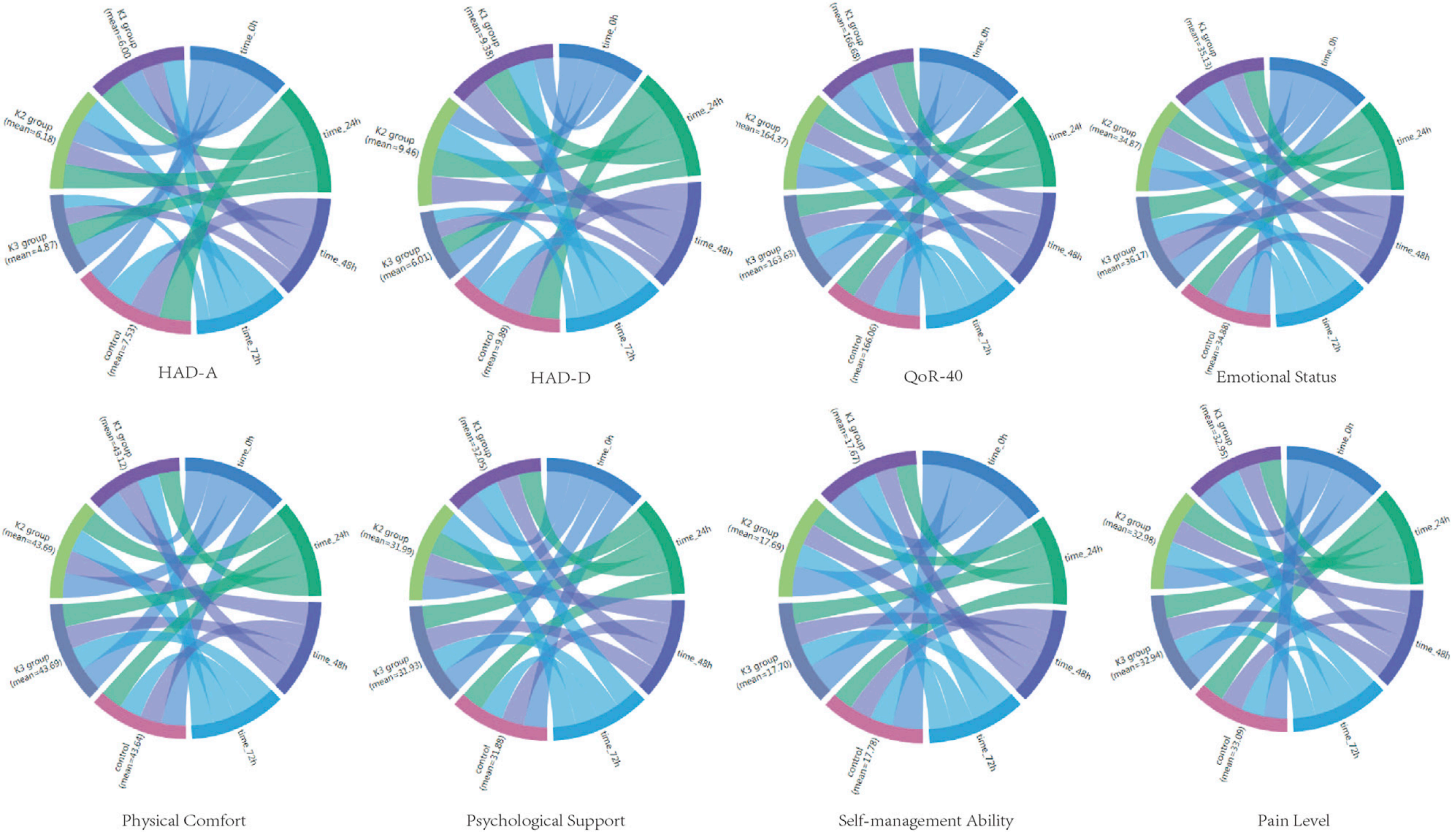
Sankey diagram of HAD-A, HAD-D, QoR-40, emotional status, physical comfort, psychological support, self-management ability, and pain level for the four groups at the time of 0, 24, 48, 72 h after operation.

There was no significant difference in the total score of HAD-A among the four groups before operation (*p* > 0.05). The total score of HAD-A in the K3 group was significantly lower than that in the C group 24, 48, and 72 h post-operation (*p* < 0.05). The total score of HAD-A in the C group was higher than that before operation, but without a significant difference (*p* > 0.05) (Table 2; Figure 2).

**TABLE 2:** HAD, QoR-40, and emotional, physical, and psychological evaluation among colorectal cancer patients.

Time, mean (SD)	K1 group (*n* = 26)	K2 group (*n* = 26)	K3 group (*n* = 26)	Control group (*n* = 26)	F	P	F	P′	t	P1	t	P2	t	P3
HAD-A														
Time_0 h	5.9 (3.4)	6.1 (3.9)	6.2 (2.9)	6.3 (3.9)	0.043	0.988								
Time_24 h	6.5 (3.3)	6.4 (3.2)	5.5 (2.2)^a^	8.3 (4.7)	2.984	0.035	4.307	0.007	−1.605	0.141	−1.717	0.064	−2.183	0.002
Time_48 h	6.0 (3.2)	6.2 (2.9)	4.4 (1.7)^a^	8.0 (4.9)	5.065	0.003	5.539	0.001	−1.793	0.122	−1.680	0.134	−2.044	0.000
Time_72 h	5.6 (3.1)	6.1 (2.4)	3.4 (1.7)^a^	7.5 (4.6)	7.341	0.000	6.700	0.000	−1.728	0.214	−1.351	0.408	−2.118	0.000
HAD-D														
Time_0 h	7.4 (3.2)	7.4 (4.9)	7.4 (3.7)	7.4 (4.0)	0.002	1.000								
Time_24 h	10.2 (4.3)	10.2 (5.0)	6.5 (4.1)^a^	11.2 (3.7)	1.129	0.000	1.343	0.000	−0.967	0.536	−0.821	0.596	−2.023	0.000
Time_48 h	10.3 (4.8)	10.3 (5.2)	5.7 (3.9)^a^ ^,^ ^b^	10.8 (3.7)	1.233	0.000	1.328	0.000	−0.423	0.955	−0.370	0.965	−2.079	0.000
Time_72 h	9.8 (4.7)	9.9 (5.4)	4.5 (3.3)^a^ ^,^ ^b^	10.1 (3.8)	1.250	0.000	1.238	0.000	−0.289	0.991	−0.118	1.000	−2.081	0.000
QoR-40														
Time_0 h	181.5 (4.7)	181.8 (8.3)	180.2 (9.0)	181.3 (8.3)	0.215	0.886								
Time_24 h	156.7 (6.6)	157.7 (6.3)	155.1 (8.0)	157.0 (9.3)	0.493	0.688	0.067	0.977	−0.145	1.000	0.276	0.979	−0.753	0.999
Time_48 h	163.7 (4.7)	158.1 (6.1)	157.9 (7.3)	161.5 (9.0)	4.268	0.007	2.214	0.091	1.083	0.600	−1.626	0.375	−1.611	0.751
Time_72 h	164.9 (5.7)	160.0 (6.9)	161.4 (9.6)	163.9 (9.6)	1.894	0.135	1.419	0.242	0.475	0.909	−1.694	0.335	−0.904	0.975
Emotion														
Time_0 h	38.4 (2.1)	37.5 (4.5)	37.2 (2.7)	38.5 (3.1)	1.144	0.335								
Time_24 h	33.3 (2.6)	33.6 (3.1)	35.7 (2.9)^a^	33.3 (2.3)	4.449	0.006	8.622	0.000	0.000	0.998	0.357	0.248	3.173	0.000
Time_48 h	34.1 (2.4)	33.9 (2.4)	35.9 (2.1)^a^	33.7 (3.0)	4.722	0.004	6.836	0.000	0.465	0.912	0.256	0.332	3.232	0.000
Time_72 h	34.7 (2.7)	34.5 (3.2)	35.9 (2.3)^a^	33.9 (2.1)	2.497	0.064	4.503	0.005	1.160	0.656	0.776	0.185	3.208	0.002
Physical comfort														
Time_0 h	46.9 (2.6)	46.7 (3.4)	46.7 (3.2)	46.6 (3.0)	0.059	0.981								
Time_24 h	41.2 (5.2)	42.5 (4.5)	42.3 (4.2)	42.4 (3.4)	0.465	0.708	0.680	0.566	−0.919	0.496	0.139	1.000	−0.036	0.999
Time_48 h	42.2 (4.9)	42.9 (3.8)	42.8 (4.1)	42.9 (4.8)	0.155	0.926	0.305	0.822	−0.513	0.734	0.032	1.000	−0.063	0.997
Time_72 h	42.2 (5.2)	42.7 (4.2)	42.9 (4.6)	42.7 (3.5)	0.155	0.926	0.288	0.834	−0.472	0.794	−0.072	0.997	0.204	0.999
Psycho comfort														
Time_0 h	32.9 (1.7)	32.7 (2.6)	32.6 (3.5)	32.5 (3.6)	0.102	0.959								
Time_24 h	31.5 (1.9)	31.7 (3.1)	31.7 (3.3)	31.4 (3.3)	0.055	0.983	0.245	0.865	0.052	0.898	0.308	0.999	0.253	0.992
Time_48 h	31.8 (2.5)	31.9 (2.8)	31.7 (3.9)	31.8 (3.1)	0.012	0.998	0.087	0.967	0.000	0.923	0.047	0.996	−0.116	0.992
Time_72 h	31.9 (2.4)	31.7 (2.9)	31.8 (4.2)	31.7 (3.3)	0.035	0.991	0.034	0.992	0.289	0.993	−0.045	0.987	0.037	1.000
Self-manage														
Time_0 h	22.7 (1.8)	22.7 (1.6)	22.5 (1.6)	22.9 (1.5)	0.160	0.923								
Time_24 h	15.1 (3.6)	15.3 (3.2)	15.1 (3.9)	15.0 (3.3)	0.028	0.994	0.076	0.973	0.080	0.988	0.299	0.945	0.077	0.967
Time_48 h	16.2 (3.7)	16.2 (3.1)	16.3 (3.2)	16.6 (2.8)	0.093	0.964	0.041	0.989	−0.466	0.990	−0.468	0.994	−0.322	1.000
Time_72 h	16.8 (3.6)	16.6 (3.6)	16.8 (3.4)	16.7 (2.7)	0.020	0.996	0.076	0.973	0.131	0.981	−0.044	0.998	0.181	0.942
Pain level														
Time_0 h	34.2 (1.2)	34.2 (1.6)	34.2 (1.2)	34.4 (1.2)	0.186	0.906								
Time_24 h	32.1 (3.7)	32.2 (3.2)	32.4 (2.9)	32.4 (3.3)	0.053	0.984	0.044	0.988	−0.318	0.996	−0.299	1.000	−0.090	0.996
Time_48 h	32.6 (3.1)	32.7 (2.1)	32.5 (2.4)	32.7 (2.9)	0.031	0.993	0.061	0.980	−0.140	1.000	0.055	0.961	−0.208	0.999
Time_72 h	32.9 (2.4)	32.9 (2.0)	32.7 (2.2)	32.9 (2.3)	0.023	0.995	0.081	0.970	0.000	0.985	0.065	0.929	−0.185	0.994

P: *p* value for the difference among groups of original data. P′: *p* value for the difference among groups of the index changes between different time point. P1: *p* value for the K1 group and control group. P2: *p* value for the K2 group and control group. P3: *p* value for the K3 group and control group.

a K3 group versus control group (*p* < 0.05).

b Versus baseline (*p* < 0.05).

SD, standard deviation; K1 group, 0.1 mg kg^−1^ ketamine group; K2 group, 0.2 mg kg^−1^ ketamine group; K3 group, 0.3 mg kg^−1^ ketamine group; HAD, Hospital Anxiety and Depression Scale; HAD-A, anxiety score of HAD; HAD-D, depression score of HAD; QoR-40, Quality of Recovery-40.

#### Changes of HAD-D in the Four Groups

There was no significant difference in the total score of HAD-D among the four groups before operation (*p* > 0.05). The total score of HAD-D in the K3 group was significantly lower than that in the C group (*p* < 0.05). The total score of HAD-D in the C group 24, 48, and 72 h post-operation was significantly higher than that before operation (*p* < 0.05) (Table 2; Figure 2).

#### Changes of QoR-40 in the Four Groups

There was no significant difference in the total score of QoR-40 among the four groups before operation (*p* > 0.05). The total score of QoR-40 in the four groups decreased 24, 48, and 72 h post-operation, but with no significant difference among the four groups (*p* > 0.05) (Table 2; Figure 2).

The emotional state scores of the QoR-40 in the K3 group 24, 48, and 72 h post-operation were significantly higher than those in the C group (*p* < 0.05).

There were no significant differences in physical comfort, psychological support, self-care ability, or pain feeling scores among the four groups (*p* > 0.05).

#### Comparison of the Sedation Scores 30 min After Extubation and at the Time of Out of the Recovery Room in the Four Groups

There was no significant difference in VAS score or sedation score among the four groups at 30 min after extubation (*p* > 0.05) (Table 3).

**TABLE 3:** Postoperative evaluation of colorectal cancer patients in the four groups.

Variable	K1 group (*n* = 26)	K2 group (*n* = 26)	K3 group (*n* = 26)	Control group (*n* = 26)	Chi-square	*p* value
SS 30 min after extubation, median (IQR)	2 (2.0–2.0)	2 (2.0–2.0)	2 (2.0–2.0)	2 (2.0–2.0)	1.032	0.794
SS at the time of out of the operation room, median (IQR)	2 (2.0–2.0)	2 (2.0–2.0)	2 (2.0–2.0)	2 (2.0–2.0)	3.635	0.304
SS difference between the two evaluation time, median (IQR)	0 (0.0–0.0)	0 (0.0–0.0)	0 (0.0–0.0)	0 (0.0–0.0)	3.037	0.386
VAS 30 min after extubation, median (IQR)	2.0 (1.8–3.0)	2.0 (2.0–2.3)	2 (1.8–2.0)	2 (2.0–3.0)	5.107	0.164
VAS at the time of out of the operation room, median (IQR)	2 (2.0–2.0)	2 (2.0–2.0)	2 (2.0–2.0)	2 (2.0–2.3)	4.957	0.175
VAS difference between the two evaluation time, median (IQR)	0 (−1.0–0.3)	0 (-0.3–0.0)	0 (0.0–0.3)	0 (−1.0–0.0)	3.305	0.347

K1 group, 0.1 mg kg^−1^ ketamine group; K2 group, 0.2 mg kg^−1^ ketamine group; K3 group, 0.3 mg kg^−1^ ketamine group; SS, sedation score; IQR, interquartile range; VAS, visual analog scale.

#### Changes of the Levels of IL-6, IL-8, and TNF-α in Peripheral Blood

**FIGURE 3:**
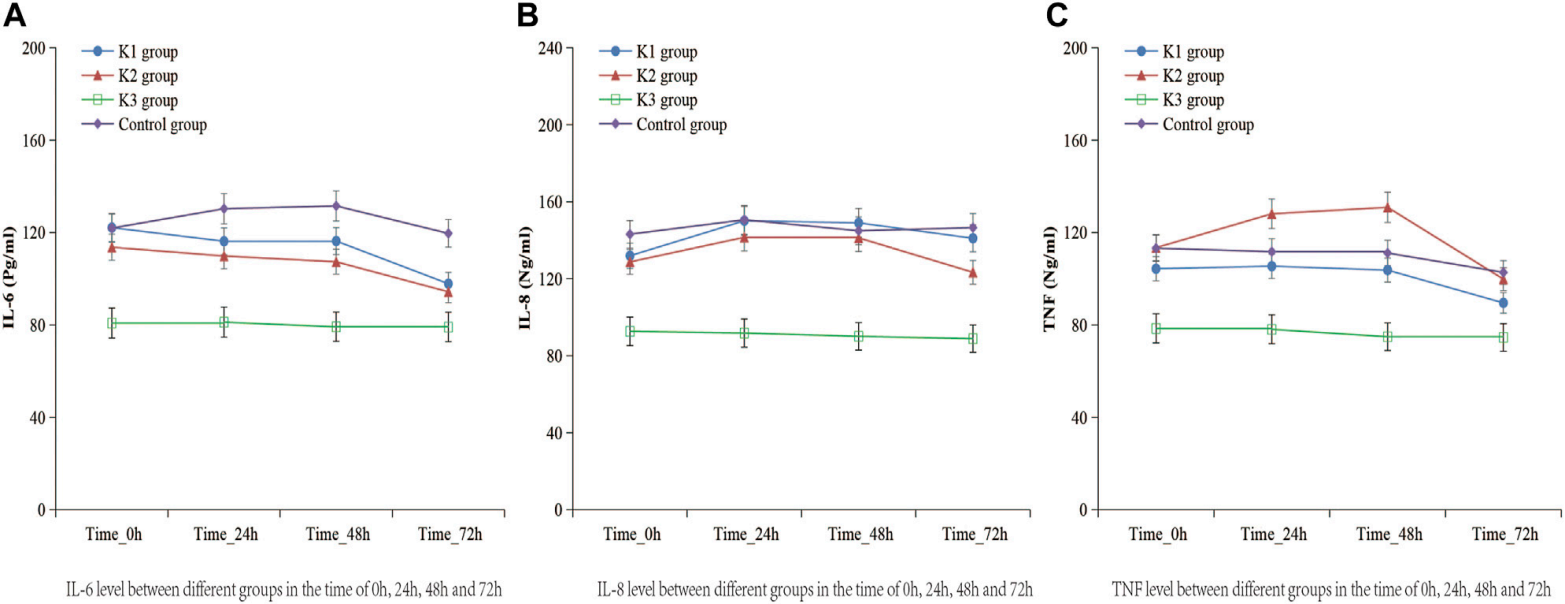
Levels of IL-6, IL-8, and TNF-α in the four groups at the time of 0, 24, 48, 72 h after operation. **(A)** K1 group, 0.1 mg kg^−1^ ketamine group; **(B)** K2 group, 0.2 mg kg^−1^ ketamine group; **(C)** K3 group, 0.3 mg kg^−1^ ketamine group.

There were no significant differences in the serum levels of IL-6, IL-8, or TNF-α among the four groups before operation (*p* > 0.05) (Table 4). The levels of IL-6, IL-8, and TNF-α in the K3 group were significantly lower than those in the C group (*p* < 0.05 and *p* < 0.01) at the end of operation, 24, 48 and 72 h after operation (Table 5; Figure 3).

**TABLE 4:** IL-6/IL-8/TNF levels before operation among colorectal cancer patients in the four groups.

Cytokines	K1 group (*n* = 26)	K2 group (*n* = 26)	K3 group (*n* = 26)	Control group (*n* = 26)	F/chi-square	*p* value
IL-6, mean (SD)	127.4 (68.7)	128.7 (73.7)	120.8 (88.5)	127.5 (65.6)	0.059	0.981
IL-6, median (IQR)	123.0 (52.5–180.0)	113.5 (63.8–171.3)	108.0 (49.8–166.5)	120.1 (73.5–164.1)	1.021	0.796
IL-8, mean (SD)	134.6 (69.5)	127.4 (52.4)	119.2 (57.4)	136.5 (53.3)	0.466	0.707
IL-8, median (IQR)	140.0 (75.3–188.8)	119.0 (78.3–159.0)	110.0 (71.8–158.3)	135.0 (88.0–168.5)	2.116	0.549
TNF, mean (SD)	114.4 (64.5)	120.1 (66.1)	107.7 (62.9)	112.9 (61.1)	0.168	0.918
TNF, median (IQR)	107.0 (65.8–140.3)	117.5 (57.8–178.5)	97.5 (61.0–160.3)	93.0 (70.3–131.0)	0.661	0.882

**TABLE 5:** IL-6/IL-8/TNF levels after operation among colorectal cancer patients in the four groups.

Cytokines and time	K1 group (*n* = 26)	K2 group (*n* = 26)	K3 group (*n* = 26)	Control group (*n* = 26)	F	P1	F	P2	F	P3
IL-6, mean (SD)					7.046	0.009	1.625	0.188	4.134	0.008
Time_0 h	122.1 (57.7)	113.6 (57.8)	80.8 (40.8)^a^	121.9 (56.4)						
Time_24 h	116.2 (57.1)	109.8 (58.9)	81.2 (35.9)^a^	130.3 (61.5)						
Time_48 h	116.3 (56.9)	107.3 (57.9)	79.2 (33.3)^a^	131.5 (55.3)						
Time_72 h	97.8 (52.4)	94.3 (50.1)	79.1 (38.8)^a^	119.6 (58.2)						
IL-8, mean (SD)					0.001	0.980	0.519	0.670	6.924	0.000
Time_0 h	131.9 (52.9)	128.7 (43.9)	92.7 (34.3)^a^	143.1 (50.9)						
Time_24 h	150.1 (62.6)	141.5 (50.2)	91.8 (37.1)^a^	150.6 (56.6)						
Time_48 h	149.0 (75.6)	141.2 (54.1)	90.1 (37.1)^a^	144.9 (53.9)						
Time_72 h	141.0 (72.4)	123.3 (45.1)	88.9 (37.1)^a^	146.5 (67.4)						
TNF, mean (SD)					7.780	0.006	0.311	0.817	2.745	0.047
Time_0 h	104.3 (61.9)	113.4 (58.1)	78.5 (38.8)^a^	113.2 (59.9)						
Time_24 h	105.4 (59.7)	128.1 (75.6)	78.1 (36.7)^a^	111.7 (58.6)						
Time_48 h	103.7 (61.1)	130.9 (80.7)	74.9 (37.5)^a^	111.1 (64.8)						
Time_72 h	89.5 (67.2)	99.8 (60.8)	74.6 (42.2)^a^	102.7 (65.9)						

P1: *p* value for time effect, P2: *p* value for the interaction between time and treatment, P3: *p* value for treatment effect.

a The difference between the K3 group and control group was statistically significant (*p* < 0.05).

### Safety Analysis

There were no significant differences in extubation time, postoperative cough, emergence agitation or delirium among the four groups (*p* > 0.05) (Table 6). No dizziness, nausea, vomiting, diplopia, or other adverse reactions were found 30 min after extubation.

**TABLE 6:** Postoperative evaluation of colorectal cancer patients in the four groups.

Variables	K1 group (*n* = 26)	K2 group (*n* = 26)	K3 group (*n* = 26)	Control group (*n* = 26)	F/chi-square	*p* value
Anesthesia extubation time (minutes), mean (SD)	11.6 (5.5)	11.9 (6.4)	11.9 (5.8)	11.9 (5.8)	0.018	0.997
Cough after extubation, n (%)					0.495	0.920
Yes	21 (80.8)	20 (76.9)	21 (80.8)	22 (84.6)		
No	5 (19.2)	6 (23.1)	5 (19.2)	4 (15.4)		
Dysphoria, n (%)						
At 30 min after extubation	3 (11.5)	3 (11.5)	2 (7.7)	1 (3.8)	1.338	0.720
At time out of the operation room	2 (7.7)	2 (7.7)	1 (3.8)	1 (3.8)	0.707	0.871
Delirium, n (%)						
At 30 min after extubation	2 (7.7)	1 (3.8)	1 (3.8)	0 (0.0)	2.080	0.556
At time out of the operation room	0 (0.0)	0 (0.0)	0 (0.0)	0 (0.0)	0.000	1.000
Disgusting, n (%)						
At 30 min after extubation	1 (3.8)	0 (0.0)	0 (0.0)	1 (3.8)	0.000	0.564
At time out of the operation room	0 (0.0)	0 (0.0)	0 (0.0)	0 (0.0)		1.000
Vomiting, n (%)						
At 30 min after extubation	0 (0.0)	0 (0.0)	0 (0.0)	0 (0.0)	0.000	1.000
At time out of the operation room	0 (0.0)	0 (0.0)	0 (0.0)	0 (0.0)	0.000	1.000

SD, standard deviation; K1 group, 0.1 mg kg^−1^ ketamine group; K2 group, 0.2 mg kg^−1^ ketamine group; K3 group, 0.3 mg kg^−1^ ketamine group.

## Discussion

Our study showed the total score of HAD-A and HAD-D in the K3 group was significantly lower than that in the C group at the pre-defined visit time (24, 48, and 72 h post-operation), which illustrated that a single sub-anesthetic dose (0.3 mg kg^−1^) of ketamine can significantly improve the postoperative anxiety and depression of colorectal cancer patients. For the C group, the postoperative score of HAD-D not HAD-A was significantly higher than that before operation (*p* < 0.05), suggesting colorectal surgery induced depression to some extent. This finding was consistent with the result of Medeiros’s study (Medeiros et al., 2010). Postoperative depression is very common in this sort of patients probably due to the immune mechanisms involved in the pathophysiological processes, together with the preoperative fear and worry, the obvious change of body structure after operation and the loss of normal defecation function.

Traditional antidepressants with drawbacks, such as long onset time, poor treatment effect, and high recurrence rate, could not meet the demands of perioperative patients to relieve depression and anxiety symptoms timely (Ibrahim et al., 2011). Ketamine has definite antidepressant effect (Hasselmann, 2014), with fast onset, 1-week lasting effect, and good effect on refractory depression (Iadarola et al., 2015). In this study, we observed that a single sub-anesthetic dose of ketamine (0.3 mg kg^−1^) by intravenous administration before operation can reduce the symptoms of depression and anxiety 24, 48, and 72 h post-operation. Mainly because the sub-anesthetic dose of ketamine can increase free glutamate by blocking NMDA receptor and further activate α-amino-3-hydroxy-5-methyl-4-isoxazolyl propionic acid receptor (AMPA) receptor and mTOR signaling pathway, which lead to the increase of synaptic protein signal level and quantity in prefrontal cortex (Li et al., 2010; Zanos et al., 2016). On the other hand, it can enhance the expression of brain-derived neurotrophic factor (BDNF) in hippocampus (Liu et al., 2012), so as to have a rapid antidepressant effect. Furthermore, alterations of GABA and 5-HT release also contributed to the fast antidepressant effects of sub-anesthetic doses of ketamine. A PET study in monkeys indicated that sub-anesthetic doses of ketamine infusion transiently increased serotonin levels in the extracellular fluid of the prefrontal cortex, selectively enhancing serotonergic transmission by inhibition of serotonin transporter activity (Yamamoto et al., 2013). Clinical studies demonstrated fast antidepressant effect of ketamine impacted the GABAergic system by reversing the decreased cortical GABA concentration and agonism of GABAA-R in patients with depression (Pham and Gardier, 2019). In addition, ketamine also has the effects of inhibiting stress, preventing postoperative pain (Moon et al., 2018), reducing inflammatory response (Yang et al., 2015), playing a long-term and whole process of preventive analgesia during and after operation, and preventing the extension of postoperative pain signal, so as to reduce the degree of depression of patients (Loftus et al., 2010).

In recent years, studies have confirmed that immune abnormalities are mediated by inflammatory cytokines in patients with depression (Patas et al., 2018), indicating that inflammatory cytokines may be involved in the pathological process of depression, which is one of the causes of depression. Surgery and stress can activate the peripheral immune system, lead to the release of inflammatory cytokines, and further activate the inflammatory response of the central nervous system. After the cytokine signal reaches the brain, it will affect the synthesis, release, and reuptake of emotion related neurotransmitters, and then induce the progression of postoperative depression.

TNF-α is an important initiation factor mediating the inflammatory response. By promoting the release of IL-6, IL-8, and other cytokines, it aggravates the damage in central nervous system. The increased expression of TNF-α can induce changes in brain structure and function, leading to the development of depression (Uint et al., 2019). IL-6 is directly related to the surgery, and the increased degree of the IL-6 level and the duration of the surgery are consistent with the degree of surgical trauma. It is a sensitive marker of tissue damage, especially reflecting the severity of inflammatory reaction (Gebhard et al., 2000). In this study, we observed that the levels of serum inflammatory factors (IL-6, IL-8, and TNF-α) were higher in the four groups, and they were significantly lower in the K3 group than those in the C group at the end of the operation, 24, 48 and 72 h after operation, indicating that a single sub-anesthetic dose of ketamine (0.3 mg kg^−1^) could effectively reduce the expression of inflammatory factors and the release of pro-inflammatory cytokines, and alleviate the nerve injury caused by inflammatory reaction (Wang et al., 2017), which mainly worked through antagonizing NMDA receptor, inhibiting nitric oxide synthase (NOS), reducing the production of nitric oxide (NO), thereby inhibiting the release of inflammatory factors (Fan et al., 2016), reducing the neuroinflammatory response, and achieving the neuroprotective effect. Other studies have found that the phenolic structure in ketamine may play a role in scavenging oxygen free radicals, inhibiting the activation of leukocytes in inflammatory reaction, reducing the levels of IL-6, IL-8, and TNF-α (Chen et al., 2018), and playing a neuroprotective role. Therefore, ketamine may be more suitable for the treatment of perioperative patients with depression and anxiety. However, ketamine may activate limbic system, so it often induces a variety of psychosis-like symptoms and cognitive impairment, such as nightmares, hallucinations, delirium, convulsions, and has a positive correlation with the dosage of ketamine, which limits its clinical application (Mion and Villevieille, 2013). Recent studies have found that a sub-anesthetic dose of ketamine can significantly reduce the psychiatric symptoms induced by ketamine. The sub-anesthetic dose of ketamine is defined as intravenous ketamine ≤ 0.5 mg kg^−1^, usually 0.1–0.5 mg kg^−1^ (Imbellon et al., 2017). Studies have also shown that 0.3 mg kg^−1^ ketamine used in painless induced abortion anesthesia did not cause delirium, sleep talk, and other mental symptoms (Schwenk et al., 2018), so we selected three different doses of ketamine in this study for clinical observation, in order to optimize the lowest effective treatment dose.

In this study, the total score of QoR-40 of colorectal cancer patients in the four groups decreased at 24, 48, and 72 h post-operation, which was consistent with the recovery process of patients after operation, with no significant difference between the ketamine group and the control group, indicating that a sub-anesthetic dose of ketamine did not affect the recovery process of patients after operation. The QoR-40 score of the ketamine group was higher than that of the control group 24, 48, and 72 h post-operation, indicating that the antidepressant effect and postoperative analgesic effect of 0.3 mg kg^−1^ ketamine can significantly improve the emotion of patients. However, the QoR-40 score of the ketamine group did not show obvious advantage in pain, which may be related to the use of analgesia pump in the four groups.

It was also observed that there were no significant differences in sedation score, choking cough, or postoperative agitation among the four groups after extubation. Few patients in the control group had moderate and severe pain (VAS>4) 30 min after extubation, so there was no obvious postoperative agitation. It was expected that ketamine could reduce the stress response during skin incision, reduce or block the hyperalgesia caused by central sensitization by regulating the polysynaptic conduction of spinal cord neurons, and increase the response of sensory center to peripheral noxious stimulation, which may significantly reduce the pain (Kawamata et al., 2000). However, the results showed that there was no significant difference in VAS score among the four groups 30 min after extubation, which may be due to the use of analgesia pump in the four groups or the small sample size. There were no significant differences in the time of extubation or the incidence of nausea, vomiting, diplopia, and hallucination between the 0.3 mg kg^−1^ ketamine group and the control group, indicating that 0.3 mg kg^−1^ ketamine did not affect the recovery of postoperative respiration or consciousness, and there was no obvious adverse reaction.

The limitation of this study is that only three dose levels of single intravenous injection of ketamine were observed in the colorectal surgery. Additional studies should be carried out to determine the optimal dose levels and administration methods/protocols. In this study, there were no significant differences in the response of the ketamine group in the postoperative extubation time, VAS score, sedation score, or cough restlessness during recovery period compared with the control group, indicating that there were no significant differences between the ketamine group and the control group in respiratory and conscious recovery, or the tolerance to tracheal catheter. The effect of analgesia pump on postoperative pain and the possibility of insufficient sample size may result in no obvious differences between the two groups.

In conclusion, a single sub-anesthetic dose of ketamine (0.3 mg kg^−1^) by intravenous administration before operation with general anesthesia can reduce the symptoms of depression and anxiety of colorectal cancer patients after operation, reduce the levels of IL-6, IL-8, and TNF-α, and improve the quality of postoperative recovery without obvious adverse reactions.

## Data Availability

The raw data supporting the conclusion of this article will be made available by the authors, without undue reservation.
